# Simulation as a Platform for Development of Entrustable Professional Activities: A Modular, Longitudinal Approach

**DOI:** 10.7759/cureus.11098

**Published:** 2020-10-22

**Authors:** Dana J Herrigel, Colleen Donovan, Elizabeth Goodman, Archana Pradhan, Mary M Bridgeman, Lauren Hogshire, Christine Fanning, Anastasia Whitman, Michael Maniaci, Sarang Kim

**Affiliations:** 1 Hospital Internal Medicine, Mayo Clinic Jacksonville, Jacksonville, USA; 2 Emergency Medicine, Rutgers Robert Wood Johnson Medical School, New Brunswick, USA; 3 Pediatrics, Rutgers Robert Wood Johnson Medical School, New Brunswick, USA; 4 Obstetrics and Gynecology, Rutgers Robert Wood Johnson Medical School, New Brunswick, USA; 5 Pharmacy Practice and Administration, Rutgers University, Ernest Mario School of Pharmacy, Piscataway, USA; 6 Pharmacy, Rutgers Robert Wood Johnson Medical School, New Brunswick, USA; 7 Hospital Internal Medicine, Princeton Plainsboro Medical Center, Princeton, USA; 8 Internal Medicine, Rutgers Robert Wood Johnson Medical School, New Brunswick, USA

**Keywords:** epa, simulation, entrustment, curriculum, medical education

## Abstract

Background

The Association of American Medical Colleges (AAMC) has recently identified a list of integrated activities to be expected of all medical school graduates entering residency: the core Entrustable Professional Activities (EPAs). Direct observation and deliberate practice of individual EPA behaviors in the clinical setting has multiple challenges, and there is limited literature describing a comprehensive, longitudinal curriculum dedicated to formative EPA assessment.

Approach

We present a model curriculum to develop and provide formative assessment of EPA skills longitudinally throughout the clinical years. Each EPA-focused training session includes a simulation case followed by several small group activities with content related to the clinical vignette in the initial simulation. We have designed this curriculum to be longitudinal and modular, and present the general framework here.

Outcomes

Step-wise implementation began in 2013. Over 450 medical students have participated in the third year (MS3) clerkship sessions, 30 in the MS4 sub-internship sessions, and over 300 thus far in the fourth year (MS4) capstone course, including students from 10 different medical schools. MS3 sessions focused on EPAs 4, 7, 8, 9, 10, and MS4 sessions had an additional focus on EPA 8. The capstone course encompassed nearly all 13 EPAs in active simulation-based learning. Opportunities to provide formative assessment through on-the-spot feedback exist throughout the curriculum. Student feedback was overwhelmingly positive.

Next steps

We found that simulations are an effective method of providing formative assessment of EPAs that are exceptionally well-received by medical students. We have demonstrated that these can be implemented for medical students from multiple educational backgrounds. We believe that deliberate practice and longitudinal formative assessment is of utmost importance in effectively developing core EPAs prior to final entrustment decisions.

## Introduction

The Association of American Medical Colleges (AAMC) has recently identified a list of integrated activities to be expected of all medical school graduates entering residency: the core Entrustable Professional Activities (EPAs) [1]. Entrustment denotes competency in specific professional activities that can be carried out without direct supervision on day one of residency [1]. It has been suggested that EPAs be incorporated into teaching in the fourth year (MS4), but formative assessment that begins earlier in clinical training allows for opportunities for feedback and improvement before entrustment decisions are made [2-7]. While EPA assessment should ideally occur in real-time clinical settings, work-place based assessment of some EPAs is often not feasible, and sometimes not ethical. For example, a medical student would not be expected to independently initiate management of a critically ill patient in most situations. 

An alternate solution is to employ simulation as a platform for developing and providing formative assessment of EPA behaviors. Simulation can reproduce clinical scenarios akin to those encountered on the wards and in the clinic, and has been effectively utilized for internship bootcamp courses [8,9]. Simulation prompts critical thinking and action by engaging the learner in dynamic interactions with a standardized patient or manikin, whereas traditional objective structured clinical exams (OSCEs) are often static. By capturing changes in patient vital signs and clinical status in response to interventions, simulation requires the learner to interact with mixed media inputs at various time points, mimicking the stochastic nature of actual clinical encounters. Previous studies have explored the use of simulation to evaluate EPAs at the end of the MS4 year, and have often focused on a single EPA, namely EPA 10 [10,11]. Our curricular framework is different as it includes numerous formative checkpoints with opportunities to encompass all EPAs, and is embedded throughout the third and fourth years of medical school.

## Materials and methods

Approach

We developed a simulation-based curriculum that spans the MS3 and MS4 years and incorporates multiple opportunities for formative assessment during each exercise. This curriculum aims to fulfill all of the elements of the 2018 Ottawa Criteria for good formative assessment [12]. The exercises included in this approach provide specific and actionable feedback that is immediate (timely) and ongoing (longitudinal). By nature, simulation is low-stakes, in that patient care is not at risk, and in our use, students were not graded but provided with immediate feedback via debriefing undertaken as a group with a skilled faculty. Formative feedback is embedded at multiple points throughout the clinical exercise. Most importantly, these exercises are intended to stimulate learning, emphasizing the catalytic effect.

This approach is designed to be modular, flexible, and generalizable. Students begin the curriculum in the MS3 year, engaging in one workshop per clerkship, typically at the midway point of the clerkship. The content of the workshop is based on a case relevant to that clerkship. We have adapted this to multiple clerkships and currently utilize this approach in four of the core clerkships (Internal Medicine, Surgery, Neurology, and Pediatrics).

Intern capstone courses have demonstrated success in improving intern practical skills on the wards [9]. Incorporation of EPAs into the capstone fourth year “bootcamp” course began in 2013. Finding success with this approach, and discovering significant gaps in undergraduate EPA focused curricula (example: prescription writing and handoffs), deliberate practice of EPAs was undertaken earlier in the clinical years. The MS3 Clerkship simulations were implemented in January 2016 at Institution one and the MS4 clerkship simulations were implemented at Institution two in July 2016. The planning committee included bootcamp course directors, MS3 and MS4 clerkship directors, and simulation directors. Institution one is a northeast medical school graduating between 160 and 190 students per year. Institution two is a clinical site of the Mayo Clinic Alix School of Medicine with over 100 visiting fourth-year students each year.

Each workshop was based on a simulation case and designed to have modular elements that may be utilized or omitted based on goals of the session for each clerkship. We utilized either a high fidelity simulator manikin voiced by a faculty facilitator, or a trained standardized patient acting as the patient in each clinical scenario. Subsequent to the clinical scenario, several small group activities ensued with content tied to the clinical vignette used in the preceding simulation.

MS3 clerkship simulations

Mid-rotation during each participating clerkship, a specialty-specific simulation was held. In the high-fidelity simulation, one faculty preceptor managed the monitor and voiced the manikin, while the other faculty preceptor acted as a nurse and subsequent debriefer. 

Students engaged in each simulation in groups of three to six. Students first participated in a 30-minute simulation, followed by a 30-minute debriefing session. For the following 15 minutes, a clinical question directly applicable to the simulated case was posed to the group, as if on rounds. The students individually developed a clinical question utilizing the PICO (Patient, Intervention, Comparison, Outcomes) framework and emailed it to the preceptor [13]. The preceptor provided immediate feedback on students’ responses. The students then sought an answer to their clinical question using electronic resources via their mobile devices. Feedback about optimal approaches for using evidence-based resources at the point of care was then provided to students. Following this exercise, the students were instructed to write the patient’s discharge prescriptions (10 minutes). They then reviewed an answer key in real time and received feedback from the preceptor. These sessions lasted 90 minutes in total, with time for movement between stations. For a typical clerkship rotation group of 24 students, six small groups of four students required three preceptors for 3.5 hours in one-afternoon session. 

Simulation cases were chosen to reflect cardinal manifestations of disease states likely to be encountered by preliminary, transitional, and categorical interns in all major specialties, such as thrombosis, bleeding, sepsis, arrhythmias, respiratory failure, encephalopathy, and acute neurologic deficit. An example simulation case is outlined in detail in Appendix A. 

Group formative feedback was provided by the faculty preceptor who observed the simulation in the clinical debriefing exercise, following the structure of good debriefing techniques [14]. Subsequent EPA exercises easily led themselves to objective checklist-style evaluations, which were immediately shared with the learners. The clinical question building/information retrieval exercise assigned points based on the student’s PICO and the resources used to answer the clinical question; however, the “grade” was intended for future research purposes, and only informal feedback was shared with the learners during the exercise. During the prescription writing exercise, debriefing was immediately undertaken, and corrections were shared with the group. Table 1 demonstrates the EPAs assessed during the simulation.

**Table 1:** Entrustable Professional Activities Mapped to Learning Modalities for the Clerkship Simulations EPA: Entrustable professional activities; EKG: Electrocardiogram; CTA: Computed tomography angiography; PICO: Patient, Intervention, Comparison, Outcomes.

EPA	EPA Description	Learning Modality
1,2,3,6,9,10	1. Gather a history and perform a physical exam 2. Prioritize a differential diagnosis following a clinical encounter 3. Recommend and interpret common diagnostic and screening tests 6. Provide an oral presentation of a clinical encounter 9. Collaborate as a member of an interprofessional team 10. Recognize a patient requiring urgent or emergent care, and initiate evaluation and management	High Fidelity Case Simulations: Team based exercises encompassing multiple EPAs: - students performed history and physical exam - students were asked by preceptor playing the role of a nurse for a differential diagnosis to guide diagnostic testing - students were asked to interpret tests and studies, such as EKG, CTA, and laboratory values - at the end of the exercise, students presented the case to the attending - students interacted with nursing staff, portrayed by a faculty member during the encounter
7	Form clinical questions and retrieve evidence to advance patient care	After simulation debrief: -students were given a clinical question tied to the SIM vignette and asked to formulate a PICO and find evidence to answer the question
4	Enter and discuss orders/prescriptions	Prescription Writing Exercise -example prescription writing: warfarin, enoxaparin

MS4 subinternship simulations

Building on this structure, near the end of the sub-internship, students on visiting rotations at Institution two engaged in a similar session. The clinical scenario was more advanced, with the simulated patient requiring more emergent care (i.e. unstable rapid atrial fibrillation in a patient with concomitant hypoxic respiratory failure.) Each session included a debrief session, formulating a clinical question using PICO, retrieval of evidence to answer the question, prescription writing, and a written handoff session. In the evening, students were called by a nurse to address a cross-cover issue, and provided with feedback on the way they managed the call. These sessions lasted 120 minutes, with additional time in the evening dedicated to nurse calls (approximately 5 minutes per call).

MS4 capstone course

The culmination of these activities manifested in the eight-day MS4 capstone course titled “Bootcamp.” The goal of the capstone course was to build an active learning curriculum that incorporated nearly all EPAs. Table 2 demonstrates the EPAs assessed during the course. The week was organized around multiple simulation cases and was structured around small groups rotating through different sessions. Similar to the preceding MS3 and MS4 sessions, high fidelity simulations were complemented by the following exercises with a greater degree of complexity tailored to graduating fourth year students: evidence retrieval, order entering or discharge prescription writing, handoffs, and nurse calls, with the addition of informed consent encounters relevant to the day’s simulation scenario. Individual and group feedback was provided in real time. The capstone course also included a procedure workshop and medical error root cause analysis module utilizing actual cases, encompassing EPAs 12 and 13. 

**Table 2:** Entrustable Professional Activities Mapped to Learning Modalities for the Capstone Course EPA: Entrustable professional activities; EKG: Electrocardiogram; CTA: Computed tomography angiography; PICO: Patient, Intervention, Comparison, Outcomes.

EPA	EPA Description	Learning Modality
1,2,3,6,9,10	1. Gather a history and perform a physical exam 2. Prioritize a differential diagnosis following a clinical encounter 3. Recommend and interpret common diagnostic and screening tests 6. Provide an oral presentation of a clinical encounter 9. Collaborate as a member of an interprofessional team 10. Recognize a patient requiring urgent or emergent care, and initiate evaluation and management	High Fidelity Case Simulations: Team based exercises encompassing multiple EPAs: - students performed history and physical exam - students were asked by a nurse or the SP for a differential diagnosis - students were asked to interpret tests and studies, such as EKG, CTA, and laboratory values - at the end of the exercise, students presented the case to the attending - students interacted with nursing staff, portrayed either by a faculty member or SP during the encounter
4	Enter and discuss orders/prescriptions	Prescription and Order Writing Exercise -example prescription writing: warfarin -example order writing: heparin infusion
7	Form clinical questions and retrieve evidence to advance patient care	Real time retrieval of clinical evidence exercise (Evidence based medicine approach using PICO methodology)
8	Give or receive a patient handover to transition care responsibility	Handover exercise – written, verbal, and simulation-based Night Nurse Calls – receiving handover
11	Obtain informed consent for tests and/or procedures	Informed Consent Simulation

Informed consent involved a flipped classroom approach to didactics and individual observed interactions with a standardized patient, based on two modules previously published on MedEdPortal by Diemer et al. in 2014 and Wathen et al. in 2011 [15,16]. Flipped classroom materials included a five-minute video modeling consent for blood transfusion and worksheets summarizing standard consent points for common procedures such as transfusion, central line placement, lumbar puncture, thoracentesis, and paracentesis. Informed consent was obtained for a blood transfusion and a bedside procedure. A unique aspect of the consent scenario was the inclusion of a “challenge statement” to allow evaluation of professionalism during these interactions. The challenge statements were based on common patient questions (i.e. “How many of these procedures have you done?” or "Doctor, have you ever seen someone have a bad reaction to a blood transfusion?" or “I cannot accept blood for religious reasons.”) Students were given direct real-time feedback on their consent approach and content, as well as their professionalism in response to the challenge statements. 

## Results

Outcomes

Students from ten different medical schools with a variety of educational backgrounds successfully participated in our simulation activities (Appendix B). Over 450 students have participated in the MS3 clerkship sessions at Institution one, 30 visiting students in the MS4 sub-internship sessions at Institution two, and over 300 thus far in the MS4 capstone course at Institution one. Despite different training backgrounds, all students were able to participate on a similar level, and found the activities highly beneficial. Formative assessment via direct observation was embedded at every point in the curriculum. 

Student feedback

Feedback from students was overwhelmingly positive and highlighted the ability of a simulation setting to provide students with a safe environment to take a leading role in attempting to manage unstable patients, as well as simply providing an opportunity to translate what they learned in the classroom into actual practice. Quintessential student feedback below represents the students’ perspective of these exercises:

"The simulation session was extremely helpful and I wish that we had more sessions like this. It was useful to have to use new skills/think emergently, without putting a real human in danger. This type of experience can't really be learned from textbooks, and so the simulation session is a very good way to practice synthesizing our book knowledge with practical skills, along with practicing team work. All I can think that would improve the session is just having more of them- I found it to be very, very useful." 

“I thought that this was a useful, realistic exercise that allowed me to think on my feet and helped prepare me for what intern year responsibilities will entail.”

"I always value simulation sessions because they foster a fun and low pressure environment to learn about management. They force critical thinking in developing differentials and integrating new information as the case progresses, and allow for discussion that doesn't always happen in the time-limited clinical setting."

## Discussion

Next steps

We present simulation as a feasible and well-received method of providing multiple opportunities for formative assessment of core EPAs throughout the MS3 and MS4 years of undergraduate medical education. We demonstrate fulfillment of the Ottawa criteria for good formative assessment, particularly the catalytic effect to stimulate learning, as described in the student evaluation comments. 

Implementation challenges include dedicating faculty time and effort, as well as simulation center availability. While high fidelity simulation with a manikin or standardized patient is ideal, low fidelity simulation (i.e. a monitor and facilitator) can be achieved on a small budget, with little detriment to the intent of the exercise. The time and resource-intensive nature of these exercises was balanced by the unique ability to deliver formative feedback based on direct observation with space and time dedicated to immediate thoughtful reflection on behalf of the students and facilitators.

Utilizing simulation as a platform allowed for direct observation of EPA behaviors and just-in-time feedback in a safe environment, mitigating risk to actual patients. Based on our experience and positive student feedback, at Institution one, we have implemented simulation even earlier in pre-clinical teaching, so simulation now takes place throughout the four years of the medical school curriculum. Consideration of a pre-subinternship bootcamp course may allow for solidification of clinical skills learned prior to real-world assessment of entrustable skills during the subinternship. 

## Conclusions

Student perception of learning value from the simulation-based activities was consistently and overwhelmingly positive. We found our curriculum to be generalizable, as we have demonstrated the feasibility of these exercises in medical students from multiple undergraduate medical schools. In summary, early adoption of EPA-based simulation curricula in the clinical years is feasible and generalizable, and lends itself to good formative assessment through catalytic learning. 

## Appendices

Appendix A: Simulation Case for MS3 Learners - Internal Medicine Clerkship

SIMULATION CASE TITLE: MS3 Internal Medicine Clerkship Entrustment Simulation: A patient with palpitations and dyspnea.

PATIENT NAME:  D.D.   PATIENT AGE: 55

CHIEF COMPLAINT: Palpitations and shortness of breath since 3 AM

Educational Objectives

1.       Recognize acutely ill patient requiring urgent care

2.       Balance history gathering with action in an acutely ill patient

3.       Initiate management of rapid atrial fibrillation

4.       Frame a differential diagnosis of new onset atrial fibrillation

5.       Recognize persistent symptoms, leading to diagnosis of PE 

6.       Initiate management of submassive PE

Equipment/Environment

❒       Manikin or Standardized patient actor - should appear alert but with increased respiratory rate and anxious.

❒       Patient monitor screen (we utilize Laerdal simulation software)

❒       Pulse oximeter

❒       Blood pressure cuff

❒       EKG leads

❒       De-identified images

❒       Nasal cannula, Ventimask, simple face mask, non-rebreather

❒       Awesome Ultrasound Simulator app (for iphone) and an iPAD to display images

Personnel

❒       One standardized patient actor or confederate to voice the manikin

❒       One confederate nurse to help guide the learners

❒       One facilitator to adjust the monitor and take notes that can be used in the debrief (may also voice the manikin)

Brief narrative description of case

A middle aged man presents with new-onset palpitations and dyspnea and is found to be in rapid atrial fibrillation due to submassive pulmonary embolism (PE). Learner goals are to initiate management of an acutely ill patient and identify precipitating cause of new onset rapid atrial fibrillation in an otherwise healthy patient.

Critical Actions

1.       Take initial vital signs and place the patient on the cardiac monitor

2.       Interpret 12 lead EKG and recognize atrial fibrillation

3.       Prescribe a dose of beta blocker or calcium channel blocker

4.       Recognize that the patient remains hypoxic and dyspneic and that further history/investigation must be undertaken

5.       Diagnose pulmonary embolism via CT angiogram

6.       Prescribe anticoagulation

7.       Present the case to the attending

8.       Escalate the patient to higher level of care

Learner Preparation/Pre-Brief

You may utilize any resources that are typically available to you in the inpatient setting, including laboratory and imaging studies, mobile resources, consultative services, and information from patient family members if available. 

Initial Presentation

Initial vital signs

GCS:15   BP:133/89   HR:174    RR:24 SpO2: 93% on room air  Temp:97.8

Overall Appearance:

The patient is a robust 55-year-old male that appears uncomfortable, tachypnic, and somewhat anxious. 

Actors and roles in the room at case start

Standardized patient actor or manikin in the role of the patient, a faculty member, or another standardized patient in the role of the confederate RN, and learners in groups of 2-6

History of Present Illness

Mr. D is a 55-year-old male with hyperlipidemia and exercise induced asthma, otherwise in good health. Several hours prior to presentation, he awoke from sleep at 3 AM with “palpitations”. He reports he felt like his heart was “pounding out of his chest”. He tried to drink some water and relax. He has been very stressed at work and attributed this sensation to stress. He felt a little better and fell back asleep. In the morning, he felt the palpitations again and shortness of breath with any exertion. He was out of breath walking to the bathroom, so he presented to the ED.

If asked on review of systems, he denies chest pain. He denies syncope, diaphoresis, nausea, vomiting, or lightheadedness. He reports associated symptom of dyspnea, worse on exertion. He has never experienced any similar prior episodes. He denies lower extremity edema, orthopnea, paroxysmal nocturnal dyspnea, weight gain, increased abdominal girth, or exertional chest discomfort or dyspnea prior to this episode. He does report feeling tired as work has been stressful. Denies focal weakness or neurologic symptoms. No cough, no fevers, chills, or sick contacts.

If asked, he was recently on a 10-hour car trip last month. No air travel. No leg pain or swelling. 

If asked, social history: Lives with girlfriend. Exercises daily - weight lifting and light cardio. No exertional dyspnea at baseline. Has been busy at work (works for the railroad in logistics) so has not exercised for about 2 weeks. 10 pack-year tobacco, quit 25 years ago. No alcohol, illicit drugs, or marijuana.

Past Medical/Surgical History

Exercise induced asthma

Hyperlipidemia - diet controlled

Hospitalized after motorcycle accident 1 year ago. He was in the hospital for several weeks and required reconstructive surgery of his face and tibia. Completely recovered after stay at rehab

If asked, he did have a blood clot during that hospital stay, it was in his arm where they had placed a PICC line. Took coumadin for 3 months. No other prior VTE history.

Medications

Albuterol prn (uses once every couple of months)

Zyrtec

Allergies

Sulfa drugs - anaphylaxis

Family History:

mother alive, 84, hypertension. Father passed away from accident, no family history of VTE or early cardiac disease

Physical Examination

General: Acute distress, appears anxious and tachypnic

HEENT: No significant findings, mucous membranes moist

Neck: No elevation of JVP noticeable

Lungs: Clear to auscultation bilaterally

Cardiovascular: S1 S2 irregularly irregular, tachycardic, no murmurs, rubs, or gallops

Abdomen: No significant findings, benign

Neurological: CN II - XII intact, strength 5/5 bilateral upper and lower extremities, no pronator drift, cerebellar intact

Skin: No rash

GU: N/A

Psychiatric: appears anxious

**Table 3:** Instructor Notes, Changes, and Case Branch points

Instructor Notes - Changes and CASE Branch Points		
Intervention / Time point	Change in Case	Additional Information
Beginning of case: Identify self and patient, establishes roles, obtains initial vital signs Initial History and physical exam	Initial vital signs with heart rate 160-170	If VS not obtained immediately, RN prompts - Doctor, do you want to get some initial vitals while you perform your history?
3 minutes: Recognition of unstable vital signs (tachycardia) Requests 12 lead EKG Oxygen saturation at 92%	EKG with rapid atrial fibrillation	RN prompts - Doctor, what rhythm is that on the EKG? His heart rate seems very fast. Should we do something about that?
Students recognize arrhythmia, need for urgent management, discuss possible interventions. Note oxygen saturation is dropping, place patient on nasal cannula Oxygen saturation at 91%	Upon dose of IV beta blocker or calcium channel blocker, patient converts to sinus tachycardia on the monitor. If adenosine given, patient will momentarily slow down then resume rapid atrial fibrillation.	If students do not recognize rhythm as atrial fibrillation, facilitator varies heart rate on monitor widely – from 120-180, creating a clearly irregularly irregular pattern on the monitor. IF students have not already requested IV access and order an IV medication, RN prompts them to ask for IV access.
After appropriate medication given, sinus rhythm established on the monitor. Patient states: Doc, I feel much better, that pounding in my chest has gone away. But I still can’t catch my breath! What is going on? Oxygen saturation has slowly decreased over the past several minutes, now at 87%	Learners recognize that patient is still symptomatic. Learners will order and interpret other tests: Chest Xray, repeat EKG, BNP and troponin, CBC, renal profile	RN prompts students to order studies that may be useful (i.e. CXR) if not requested. RN also prompts that oxygen saturation has now decreased. Suggests supplemental oxygen.
O2 saturation 94% on nasal cannula (2-4 L) HR 115, sinus tachycardia, patient becomes more visibly tachypnic and dyspneic.	Students receive additional studies: chest xray (clear lungs), troponinT 0.03, NT-proBNP 848 TSH 3.0 Free T4 1.1 Na: 136 K 3.6 CL:99 HCO3: 24.8 BUN 10 Cr 1.0 Glu 82 INR 1.0 PTT 36 Ddimer – 1024 (Ddimer units) ABG: 7.44 / 29 / 70 / 94% on 4L NC CXR - clear UDS - negative	
Students discuss and interpret studies, usually think of PE and request additional studies to confirm diagnosis	Duplex: L common femoral DVT (only available if they request bedside ultrasound) Echo: Normal EF, Enlarged RV, mildly hypokinetic Mod-severe TR RVSP 50 (again only available if they request bedside point of care or STAT echo) VQ --> unavailable, nuclear medicine busy with a bleeding scan	
When CTA is ordered, RN prompts, “I don’t feel comfortable taking him to CT unless one of you will come with me.”	Students may order empiric dose of anticoagulation, or agree to go with nurse to CTA.	CTA – show images. They have to call radiology for urgent read if they cannot interpret on their own.
Students recognize PE and request IV anticoagulation with LMWH or heparin infusion	Nurse reports the attending is on the phone requesting a presentation of the patient.	Students report to attending (preferably not the dominant student, choose to give the phone to someone who has been more passive in the scenario to check their understanding). Student requests upgrade of patient to ICU or intermediate care unit for close hemodynamic monitoring.

Ideal scenario flow

Students enter and assign roles: one to take history, another to take notes, another to obtain vital signs and watch the monitor, another to examine patient. Two large bore IV access is requested. Initial history obtained and brief focused exam conducted to evaluate CV, lungs, neck veins, abdomen, and extremities, assessing for edema and pulses. Once vitals are available, learners request 12 lead EKG and interpret as atrial fibrillation, discuss management strategies and decide on rate control. Metoprolol 5 mg IV push or weight based bolus diltiazem are both acceptable choices for initial management. Once given, patient converts to sinus rhythm, but learners note that he is still quite symptomatic, and oxygen saturations are dropping. CXR and bloodwork including troponin and BNP (but not DDimer!) are requested. Patient is placed on nasal cannula, then 100% non-rebreather if necessary. Students request further history including focused review of systems and social history to rule out drug or alcohol abuse. At this time, usually discover that he has recently traveled and has a prior history of PICC-associated DVT. Chest X-ray is interpreted as clear. Students may request bedside point-of-care ultrasound at this time and note enlarged, hypokinetic RV and bilateral A-lines on lung exam. If not, students request CTA and discover bilateral proximal PE. Students initiate anticoagulation (rationale exists for Lovenox or heparin infusion as initial management, as this is classified as submassive PE). The scenario ends with students presenting the patient to their attending on the phone or in person. 

Variant for more advanced learners (MS4)

A variant of this case for more advanced learners involves the patient presenting with hypotension in the setting of rapid atrial fibrillation. The students at this point must consider and prepare for cardioversion, although the patient does not always require cardioversion as vitals will stabilize with a small fluid bolus or small dose of beta blocker or amiodarone. The remainder of the case ensues as above but patient remains with borderline hypotension, prompting a discussion of potential thrombolysis. The subsequent debrief focuses more on the evidence behind management of submassive and massive PE rather than basic management of critically ill patient. 

Anticipated management mistakes

1.       History taking too extensive without obtaining vital signs or placing patient on the monitor - we found that our students were accustomed to standard history taking in an OSCE format, where they leisurely performed a complete history, and subsequently a complete physical exam. The point of this case is to balance urgent management with history taking in a patient requiring immediate care. The RN will prompt the students to obtain vitals and recognize the gravity of the situation if they do not immediately.

2.       We also noted difficulty with placement and interpretation of bedside monitors - RN assists with this skill (when requested) and we include a brief tutorial on bedside monitors in the debrief prior to leaving the room.

3.       Misrecognition of atrial fibrillation - The RN should prompt the students to COMMIT to naming the rhythm on the EKG. If students are having trouble, the facilitator widely varies the rate from 110’s to 180 on the monitor to emphasize the irregularity of the arrhythmia. Occasionally MS3 students will request cardioversion, at which point the RN indicates that the blood pressure remains stable, and encourages them to frequently recheck the blood pressure. If adenosine is ordered, the patient’s monitor will show fibrillation waves for 2-3 seconds and then resume rapid atrial fibrillation. This can be demonstrated by having the facilitator switching the monitor into ventricular fibrillation for 2 seconds only and then back into atrial fibrillation, while the RN or standardized patient remains clearly awake and in no more distress than they were before the adenosine was administered.

4.       If students choose to give adenosine, they will often not request continuous 12 lead EKG. We correct this by mentioning it in the debrief. A continuous 12 lead rhythm strip must be recorded during the administration of adenosine.

5.       Students often request DDimer - even though in the debrief they often mention that their pre-test probability for PE was high. We reiterate the rationale for NOT ordering this test in the context of high pre-test probability in the debrief.

6.       Students often request studies that require the patient to be moved from the room and are not practical - such as sending the patient for a VQ scan or lower extremity duplex while the patient is visibly in distress and actively deteriorating. We have the nurse prompt that the VQ is not available, and when they order the CTA she requests accompanying student to be present for the study. IF they order a bedside ultrasound for DVT or point of care echo, it is allowed.

7.       Students often forget to risk stratify the patient - by ordering troponin, BNP, and echo. It is these studies which reveal the submassive/intermediate risk PE as there is both myocardial injury and dysfunction.

APPENDIX B. List of Medical Schools with Participating Students

Mayo Clinic Alix School of Medicine

Rutgers’-Robert Wood Johnson Medical School

Dartmouth Geisel School of Medicine

St. George’s University College of Medicine

University of Central Florida College of Medicine

Meharry Medical College

Morsani College of Medicine University of South Florida

Ponce School of Medicine

Nova Southeastern College of Osteopathic Medicine

Lake Erie College of Osteopathic Medicine

Florida Atlantic University College of Osteopathic Medicine

Lincoln Memorial University Debusk College of Osteopathic Medicine

Campbell University School of Osteopathic Medicine
